# Trends in the Incidence and Treatment of Early-Onset Pancreatic Cancer

**DOI:** 10.3390/cancers14020283

**Published:** 2022-01-07

**Authors:** Michael LaPelusa, Chan Shen, Nina D. Arhin, Dana Cardin, Marcus Tan, Kamran Idrees, Sunil Geevarghese, Bapsi Chakravarthy, Jordan Berlin, Cathy Eng

**Affiliations:** 1 Department of Internal Medicine, Vanderbilt University Medical Center, Nashville, TN 37232, USA; nina.arhin@vumc.org (N.D.A.); dana.cardin@vumc.org (D.C.); jordan.berlin@vumc.org (J.B.); cathy.eng@vumc.org (C.E.); 2 Department of Surgery, Penn State College of Medicine, Hershey, PA 17033, USA; cshen@pennstatehealth.psu.edu; 3 Department of Public Health Sciences, Penn State College of Medicine, Hershey, PA 17033, USA; 4 Department of Surgery, Division of Surgical Oncology and Endocrine Surgery, Vanderbilt-Ingram Cancer Center, Vanderbilt University Medical Center, Nashville, TN 37232, USA; marcus.c.tan@vumc.org (M.T.); kamran.idrees@vumc.org (K.I.); 5 Department of Surgery, Division of Hepatobiliary Surgery and Liver Transplantation, Vanderbilt University Medical Center, Nashville, TN 37232, USA; s.geevarghese@vumc.org; 6 Department of Radiation Oncology, Vanderbilt-Ingram Cancer Center, Vanderbilt University Medical Center, Nashville, TN 37232, USA; bapsi.chak@vumc.org

**Keywords:** pancreatic cancer, early-onset, SEER, incidence

## Abstract

**Simple Summary:**

Pancreatic cancer is being diagnosed more frequently in younger individuals. However, limited insight exists into the magnitude of this increase, which subgroups are most affected, and which treatments are utilized in this population. In our study, we aimed to characterize which, and how, subgroups in the United States were affected by pancreatic cancer from 2000 to 2016. Additionally, we aimed to show which therapies were used to treat young patients with pancreatic cancer. Our findings provide valuable information regarding which subgroups face higher rates of this disease and what therapies have historically been used for treatment. Clinicians, scientists, policymakers, and the general population can use this information to develop programs to educate and identify individuals who are at risk for developing pancreatic cancer at an early age, as well as to study whether younger patients should be treated differently than older patients.

**Abstract:**

Background: Early-onset pancreatic cancer (EOPC) is relatively uncommon. It is unclear if the incidence of EOPC is evolving and how these patients are treated. Methods: We conducted a retrospective, population-based study using SEER 2004–2016. We evaluated annual age-adjusted incidence rate (AAIR), stage at presentation, and race/ethnicity among 7802 patients plus treatment patterns in 7307 patients (excluding neuroendocrine tumors) younger than 50. Results: The AAIR was higher in males while the rate increased faster in females. The AAIR was highest in Non-Hispanic Black patients and increased for all races/ethnicities over time. The percentage of patients diagnosed with distant-stage disease decreased over time but increased for localized-stage disease. Hispanic patients made up a larger proportion of patients over time compared to other groups. For localized-stage disease, primary surgery alone was the most utilized modality of therapy. For regional-stage disease, chemotherapy with radiation was the most utilized modality from 2004–2010, whereas chemotherapy alone was the most utilized from 2011–2016. For distant-stage disease, chemotherapy alone was the most utilized and used increasingly over time. Patients with EOPC received radiation and chemotherapy at similar rates to, and underwent surgery more frequently, than patients 50–69. Conclusions: The AAIR of EOPC increased over time, faster so in females. Groups who experience a higher burden of pancreatic cancer, particularly African Americans, experienced a higher burden of EOPC. Treatment of localized and regional-stage disease did not follow standard treatment guidelines for pancreatic cancer. Our findings indicate that EOPC patients received more treatment than their older counterparts.

## 1. Background

In 2021, there were an estimated 60,430 new cases of pancreatic cancer. Pancreatic cancer is one of the few cancers with increasing incidence since 1998. Among all cancer types, pancreatic cancer was responsible for the third-highest number of cancer-related deaths [1,2]. From 2009–2015, pancreatic cancer had the lowest 5-year relative survival rate of all cancers, at 9% [3]. By 2030, pancreatic cancer may become the second leading cause of cancer-related deaths [4]. High rates of local infiltration, distant metastasis, and limited treated options are responsible for the high mortality rate seen in pancreatic cancer. Less than 20% of patients diagnosed are eligible for curative surgical resection [5]. 

Early-onset pancreatic cancer (EOPC) is a relatively uncommon phenomenon. From 2011–2015, overall cancer incidence rates (per 100,000) in the US for people 20–49 years old (y/o) was 115.3 for males (compared to 494.3 for males of all ages) and 203.3 for females (compared to 420.5 for all ages). The incidence of pancreatic cancer in this population was 14.6 in males and 11.2 in females [6]. From 2013–2017, 89.4% of pancreatic cancer diagnoses were in patients >55 y/o, 8.1% in patients 45–54 y/o, 1.8% in patients 35–44 y/o, and 0.7% in patients <35 y/o [7]. 

It is unclear if the incidence of EOPC is evolving. Additionally, no large studies have examined what therapies are used to treat patients with EOPC. In this study, we aim to understand the trends in incidence and treatment among patients with EOPC over the last two decades using the Surveillance, Epidemiology, and End Results (SEER) database.

## 2. Materials & Methods

We conducted a retrospective, population-based study to examine cases of EOPC using data from the SEER registry database 2004–2016 by the National Cancer Institute (NCI). The SEER cancer registry is an authoritative source for cancer statistics in the U.S. encompassing approximately 35% of the U.S. population [8]. Using SEER*Stat software we obtained the annual age-adjusted incidence rate (AAIR), defined as the annual weighted average of the age-specific (crude) incidence rate, where the weights are the proportions of persons in the corresponding age groups of a standard population [8]. Using SEER 18 custom data with additional treatment fields, which provides detailed information on tumor characteristics, demographics, treatment, and survival rates, we evaluated trends in terms of stage of presentation, race/ethnicity, and treatment pattern. SEER stages are divided into localized-stage disease, regional-stage disease, and distant-stage disease. SEER defines localized-stage disease as “limited to the organ of origin; no spread beyond organ of origin; infiltration past basement membrane of epithelium into stroma of organ. SEER defines regional-stage disease as “tumor extension beyond limits of organ of origin”, and also as, “that area extending from the periphery of an involved organ that lends itself to removal en bloc with a portion of, or an entire, organ with outer limits to include at least the first level nodal basin”. SEER defines distant-stage disease as “a tumor which has spread to areas of the body distant or remote from the primary tumor”. The statistical analyses were conducted in SAS 9.4. We evaluated the annual AAIR and trends in terms of stage of presentation and race/ethnicity among the 7802 pancreatic cancer patients <50 y/o when diagnosed. Further, we examined the treatment pattern in a subset of 7307 patients, excluding those with pancreatic neuroendocrine tumors (pNET), since the treatment of patients with pNET is different from pancreas cancer without neuroendocrine histology.

## 3. Results

The demographics of the study population are shown in Table 1. A total of 7307 young adults <50 y/o diagnosed with pancreatic cancer (excluding patients with pNET) from 2004–2016 were identified. The median age at diagnosis was 45.0 y/o. There were 3962 male patients (54.2%) and 3345 female patients (45.8%). 4175 patients were Non-Hispanic White (57.6%), 1127 patients (15.6%) were Non-Hispanic Black, and 1278 patients (17.8%) were Hispanic. 857 patients (11.7%) were diagnosed with localized-stage disease at presentation, 2243 patients (30.7%) were diagnosed with regional-stage disease at presentation, and 4207 patients (57.6%) were diagnosed with distant-stage disease at presentation. 4198 (57.5%) were diagnosed with adenocarcinoma not otherwise specified (NOS). 

**Figure 1:**
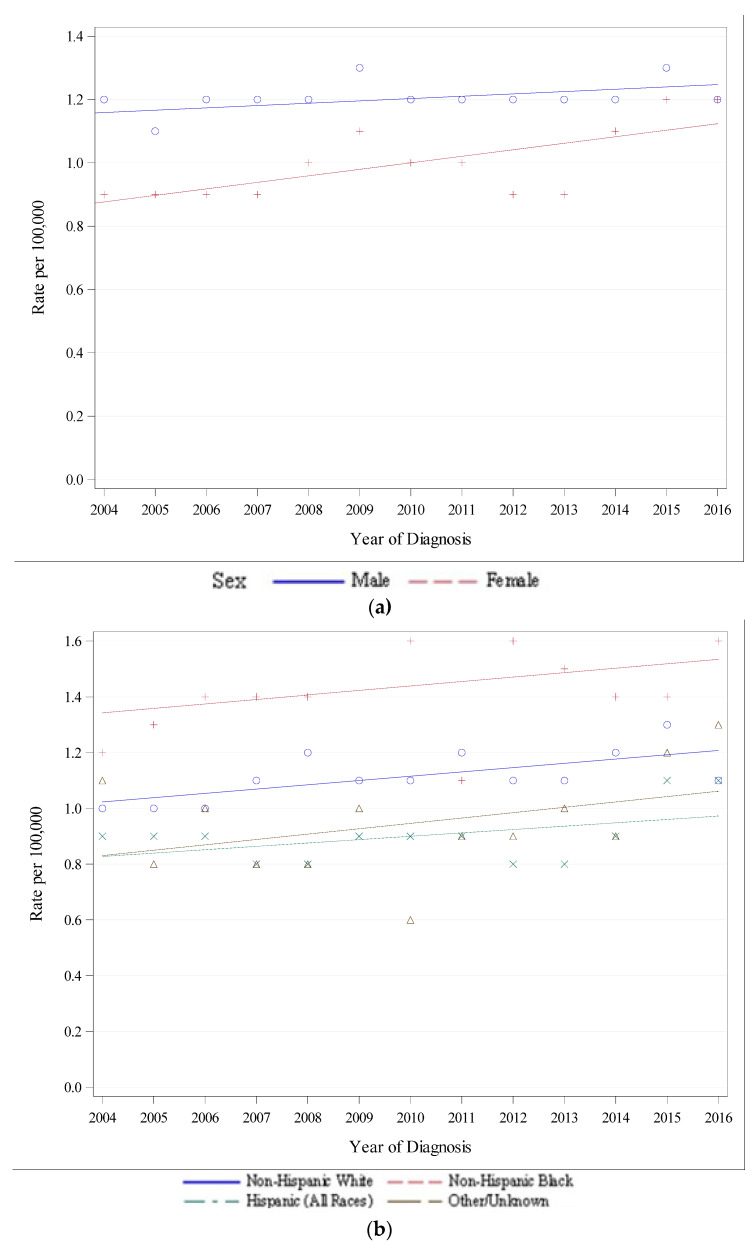
(**a**) Recent Trend in SEER-18 Age-Adjusted Incidence Rates by Sex, 2004–2016, all Races and Stages, Ages < 50, Delay-Adjusted Rate. (**b**) Recent Trend in SEER-18 Age-Adjusted Incidence Rates by Race/Ethnicity, 2004–2016, All Sexes and Stages, Ages < 50, Delay-Adjusted Rate.

Figure 1a,b show trends in the incidence of early-onset pancreatic cancer by gender and race/ethnicity, respectively. Although the AAIR was greater in males, the rate of increase was faster in females (annual percent change of 0.9 in males and 2.2 in females). The AAIR was highest in Non-Hispanic Black patients, followed by Non-Hispanic White patients. Hispanic patients had the lowest AAIR. The AAIR increased for all groups when stratified by gender and race/ethnicity, as seen in Table 2.

**Figure 2:**
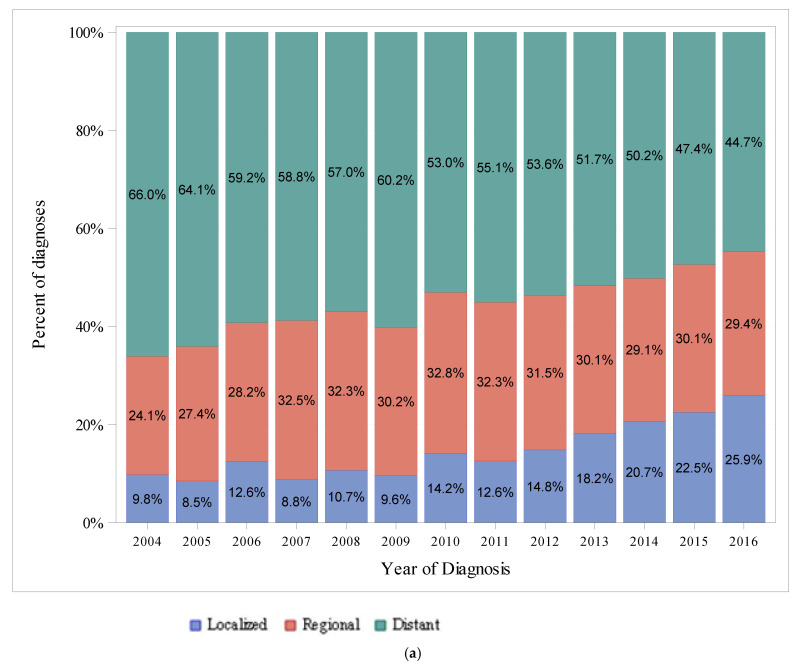

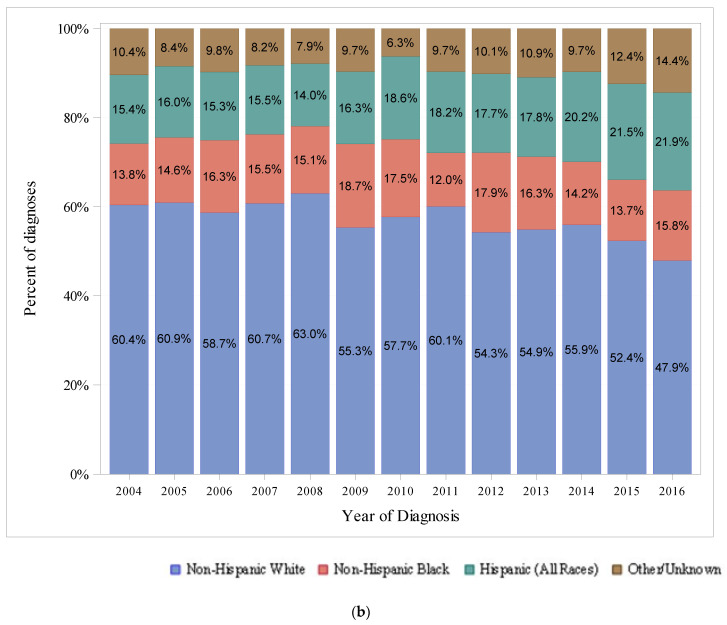
(**a**) Differences in Stages at Presentation from 2004–2016 in SEER, Ages < 50. (**b**) Differences in Race/Ethnicity from 2004–2016 in SEER 18, Ages < 50.

Figure 2a,b show trends in the percentage of diagnoses by stage at presentation and race/ethnicity, respectively. Diagnoses of distant-stage disease represented the highest percentage of diagnoses among all years in the study period, while localized-stage disease represented the lowest percentage of diagnoses among all years in the study period. The percentage of patients diagnosed with distant-stage disease decreased over time, from 66% in 2004 to 44.7% in 2016, whereas the percentage of patients diagnosed with localized-stage disease increased over time, from 9.8% of diagnoses in 2004 to 25.9% in 2016. The highest percentage of diagnoses was in Non-Hispanic White patients among all years in the study period, and the percentage of diagnoses in Non-Hispanic White patients decreased over time, from 60.4% in 2004 to 47.9% in 2016. The percentage of diagnoses in Non-Hispanic Black patients remained relatively constant over time, from 13.8% in 2004 to 15.8% in 2016. The percentage of diagnoses in Hispanic patients increased over time, from 15.4% in 2004 to 21.9% in 2016. 

**Figure 3:**
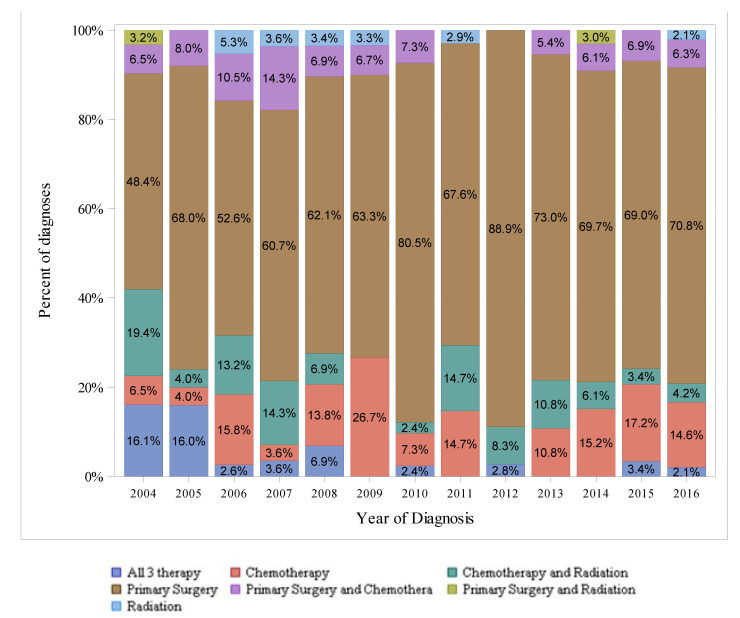
Differences in SEER-18 Localized Stage by Type of Therapy (Excluding pNET), 2004–2016, Ages < 50.

**Figure 4:**
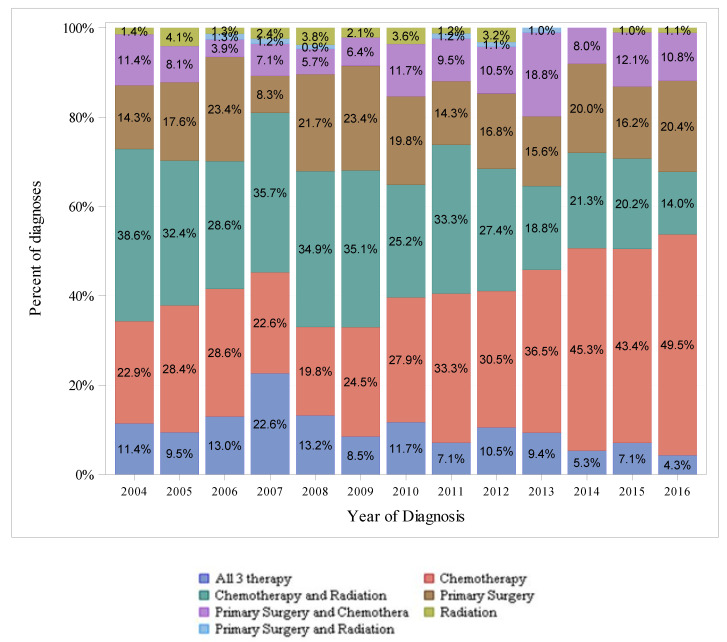
Differences in SEER-18 Regional Stage by Types of Therapy (Excluding pNET), 2004–2016, Ages < 50.

**Figure 5:**
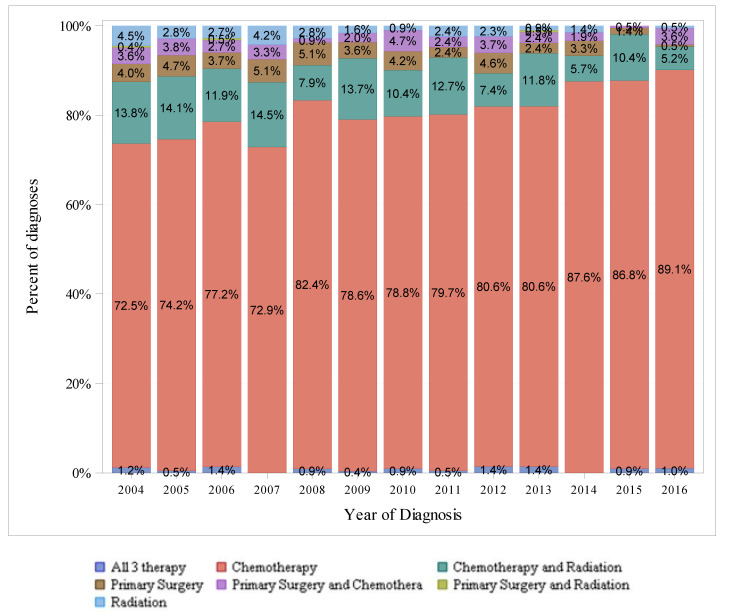
Differences in SEER-18 Distant Stage by Types of Therapy (Excluding pNET), 2004–2016, Ages < 50.

Figure 3, Figure 4 and Figure 5 show the therapeutic modalities used to treat localized, regional, and distant-stage disease, respectively. For localized-stage disease, primary surgery alone was the most frequently utilized therapy modality among all years in the study period. For regional-stage disease, combined chemotherapy with radiation was the most frequently utilized therapy regimen from 2004–2010 (utilized in 38.6% of diagnoses in 2004 to 25.2% in 2010), whereas chemotherapy alone was the most frequently utilized therapy modality from 2011–2016 (utilized in 34.3% of diagnoses in 2011 to 49.0% in 2016). For distant-stage disease, chemotherapy alone was the most utilized therapy modality among all years in the study period and was utilized in an increasing percentage of diagnoses over time (from 78.3% in 2004 to 89.1% in 2016).

Appendix A Table A1 shows treatment patterns by age group. Patients <50 y/o received radiation and chemotherapy at a similar rate compared to patients 50–69 y/o (18.5% vs. 18.1% and 61.6% vs. 60.2%, respectively) while undergoing surgery more frequently (16.8% compared to 9.5%. Patients > 70 y/o received less radiation, chemotherapy, and surgery (11.3%, 36%, and 5.7%, respectively).

## 4. Conclusions

The number of cases of EOPC each year ranged from 515 cases (7.0% of all cases) in 2014 to 634 cases (8.7% of call cases) in 2009 (Table 1), and the AAIR of EOPC increased over time (Figure 1). In the US, pancreatic cancer diagnoses are increasing [9,10]. Some have hypothesized that increasing rates of obesity in the US are contributing to the rise [11,12,13,14]. An analysis of the North American Association of Central Cancer registries showed that obesity-related cancers are increasing in the US among younger patients [15]. Others have suggested that advances in high-resolution imaging and increased use of endoscopic ultrasound are detecting more cases of early-stage disease [10]. However, the incidence of cases among all stages is increasing, implying that detection of early-stage disease may not be the main driver behind the increase in total incidence [9].

The higher overall incidence of EOPC in males in our analysis was also demonstrated in several single-institution and population-based registry analyses [10,16,17]. 

In our study, African Americans, categorized as “Non-Hispanic Black” experienced the highest AAIR of EOPC in our analysis compared to other races/ethnicities. In the US from 2001 to 2015, the incidence of pancreatic cancer among all age groups was higher among African Americans than Caucasians, at 24.7 per 100,000 vs. 19.4 per 100,000, with a higher incidence of distant-stage disease and lower incidence of localized-stage disease in African Americans compared to Caucasians [18]. One analysis of the American Cancer Society Cancer Prevention Study II found a 42% increased risk of pancreatic cancer mortality in African Americans than Caucasians from 1984–2004, which the authors concluded was not related to differences in the prevalence of risk factors, such as smoking, obesity, family history, and diabetes [19]. These findings were contrary to those found in a case-control analysis of 434 patients, which concluded that the increased risk of pancreatic cancer in African American men was secondary to higher rates of smoking, diabetes, family history, body mass index, and alcohol consumption [20]. 

Hispanic patients experienced a faster increase in AAIR than other groups in our study. The AAIR of pancreatic cancer among all age groups in Hispanic men and women decreased between 1989–2003, with a delay-adjusted annual percent change of −0.1 and −0.2, respectively [21]. Other analyses of pancreatic cancer incidence by race/ethnicity have found that Caucasians and Hispanics have a similar risk for developing pancreatic cancer, although Hispanics have a higher prevalence of obesity-related cancers [22,23]. One explanation for our findings (regarding the increasing percentage of diagnoses made in Hispanic patients) be related to the increased prevalence of childhood and adolescent obesity among Hispanics in the US, as the prevalence of obesity among US children and adolescents increased from 1999 through 2014 for adolescents 12–19 y/o—attributed “primarily by to increases in non-Hispanic Black and Hispanic youth” [24,25]. Increased rates of adolescent smoking in Hispanic youth may be another driver of our findings, as a 2017 report from the Centers for Disease Control and Prevention 13% of Hispanic youth initiated cigarette smoking <13 y/o, compared to Caucasian youth (10%) and African American youth (10.5%) of the same age between September 2016 and December 2017 [26]. 

Pancreatic cancer in the US across all age groups is diagnosed most frequently at distant-stage disease, similar to the findings in our analysis of patients with EOPC. In the US, among pancreatic cancer among all ages, the incidence of localized-stage pancreatic cancer was approximately 1 per 100,000, whereas the incidence of regional-stage disease and distant-stage disease was 3–4 per 100,000 and 7–8 per 100,000, respectively [9]. Approximately 15–20% of patients with pancreatic cancer present with resectable disease [27]. We found the percentage of patients with EOPC presenting with localized-staged disease (a loose correlate for resectable disease) was less than 15% from 2004–2012, and from 2013–2016, this percentage increased over time to a peak of 25.9%. Several single-center reviews and one population-based registry review of patients with EOPC have shown that younger patients were more likely to present with unresectable disease [28,29,30,31]. 

The National Comprehensive Cancer Network (NCCN) Guidelines for pancreatic adenocarcinoma offer evidence-based recommendations for treating pancreatic cancer. For localized and regional-stage disease, the most frequently utilized therapies in our study were not the modalities of therapies as recommended by standard treatment guidelines, which, in general, are regimens of surgery and chemotherapy for localized and regional-stage disease. Additionally, our analysis showed surgery alone (without chemotherapy) was overwhelmingly used for localized-stage disease. Chemotherapy, chemotherapy with radiation, and primary surgery alone were the most utilized therapies for regional-stage disease. One explanation for the difference from standard treatment guidelines may be that SEER staging (as described above) differs from the American Joint Committee on Cancer staging system used in clinical practice. A caveat to the interpretation of the treatment pattern for regional-stage disease is that treatment, as recommended by NCCN, differs between borderline-resectable disease (regimens primarily consisting of neoadjuvant chemotherapy and surgery) and locally advanced disease (regimens consisting of chemotherapy or chemoradiation and surgery, if appropriate). In our analysis, patients with EOPC underwent more surgery than 50–69 y/o patients and received radiation, chemotherapy, and surgery more frequently than >70 y/o patients (Appendix A Table A1). Given the higher proportion of distant-stage diagnoses in the EOPC population, where NCCN generally recommends chemotherapy alone, the higher treatment rates are notable. Other studies have also shown that patients with EOPC are treated differently than older patients. A recent analysis of 248,634 patients in the National Cancer Data Base (NCDB), a hospital-based database of patients that is not designed to be representative of the US population, found patients with EOPC receive more chemotherapy (38% vs. 29%), surgery (9% vs. 6.9%), chemoradiation (12% vs. 9.2%), and multimodal treatment (21% vs. 15%). This analysis also found that patients with EOPC had a higher one-year overall survival rate across all stages than older patients [32]. Two single-center reviews of patients with EOPC have also shown differences in the treatment of young patients compared to older patients, with younger patients generally receiving more therapy [30,33].

**Figure A1:**
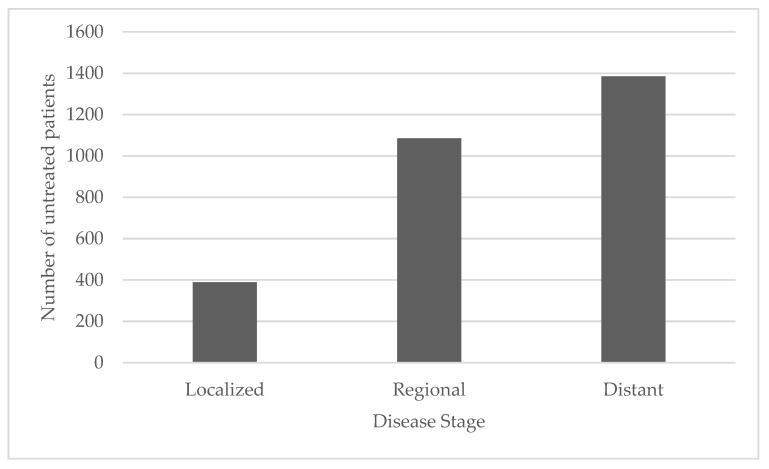
Untreated Patients with EOPC.

In conclusion, the age-adjusted incidence of EOPC is rising in the US according to our analysis of SEER. While SEER collects and publishes cancer incidence from population-based cancer registries encompassing 48 percent of the US population, it is generally regarded as an authoritative source of information on cancer in the US. Females are on track to experience rates of EOPC similar to rates in males. The same groups who suffer a higher relative burden of pancreatic cancer, particularly African Americans, experience a higher relative burden of EOPC. Research is needed to understand whether differences in the prevalence of modifiable risk factors among those most at risk for EOPC so targeted interventions can be developed. Diagnoses of localized-stage disease represented a larger proportion of diagnoses of EOPC over time. Theoretically, this could lead to higher cure rates and better survival among the EOPC population. However, the reason why EOPC was increasingly diagnosed at an early stage is unclear. Recent analyses suggest certain somatic mutations may be more frequently identified in tumors in patients with EOPC, with critical alterations in cellular pathways that are particularly targeted by smoking-associated carcinogenesis [34,35]. However, smoking rates in the US have decreased over the last two decades [36]. Further understanding of the genetic landscape of tumors in patients with EOPC is needed, especially to understand whether obesity and alcohol-related changes could play an outsized role in carcinogenesis in patients with EOPC. Additionally, campaigns should be established to educate at-risk young individuals on the risks of smoking, alcohol use, obesity, and pancreatic cancer development. Patients with EOPC receive more treatment than their older counterparts with pancreatic cancer, suggesting treatment of EOPC does not follow standard treatment guidelines. Interestingly, 2859 patients with EOPC, representing 36.6% of our study population, did not receive any treatment. This subset included 389 patients with localized-stage disease, 1085 patients with regional-stage disease, and 1385 patients with distant-stage disease (Appendix A Figure A1). The etiology for this lack of treatment is unknown, as single-center analyses of EOPC often do not include information on untreated patients. However, one study suggested that patient insurance status, comorbidity prevalence, proximity to treatment, income, and education level may contribute to lower treatment rates for patients with pancreatic cancer [32]. Additional evaluation of the population of patients with untreated EOPC is warranted to characterize the reasons for, and barriers to, not pursuing treatment.

## Figures and Tables

**Table 1:** Patient Demographics (Excluding pNET).

Demographic	Total *N* = 7307
Age	
*N*	7307
Mean (SD)	43.2 (6.53)
Median	45.0
Range	0.0, 49.0
Sex, *n* (%)	
Female	3345 (45.8)
Male	3962 (54.2)
Race/Ethnicity, *n* (%)	
Non-Hispanic White	4175 (57.6)
Non-Hispanic Black	1127 (15.6)
Hispanic (All Races)	1287 (17.8)
Other/Unknown	656 (9.1)
Marital Status, *n* (%)	
Divorced/Separated	834 (11.4)
Married (including common law)	3950 (54.1)
Single (never married)	2133 (29.2)
Unmarried/Unknown	309 (4.2)
Widowed	81 (1.1)
Year of Diagnosis, *n* (%)	
2004	570 (7.8)
2005	539 (7.4)
2006	565 (7.7)
2007	569 (7.8)
2008	587 (8.0)
2009	634 (8.7)
2010	584 (8.0)
2011	579 (7.9)
2012	559 (7.7)
2013	537 (7.3)
2014	515 (7.0)
2015	539 (7.4)
2016	530 (7.3)
Histological Classification, *n* (%)	
8000/3: Neoplasm, malignant	145 (2.0)
8010/3: Carcinoma, NOS	372 (5.1)
8140/3: Adenocarcinoma, NOS	4198 (57.5)
8480/3: Mucinous adenocarcinoma	65 (0.9)
8500/3: Infiltrating duct carcinoma,	635 (8.7)
Other types	1892 (25.9)
Stage of Presentation, *n* (%)	
Localized	857 (11.7)
Regional	2243 (30.7)
Distant	4207 (57.6)
Primary Site of Pancreatic Cancer, *n* (%)	
C25.0—Head of pancreas	3363 (46.0)
C25.1—Body of pancreas	859 (11.8)
C25.10—Other site of Pancreas	761 (10.4)
C25.2—Tail of pancreas	1331 (18.2)
C25.9—Pancreas, NOS	993 (13.6)
Beam Radiation, *n* (%)	
Yes	1332 (18.5)
None/Unknown	5857 (81.5)
Chemotherapy, *n* (%)	
No/Unknown	2808 (38.4)
Yes	4499 (61.6)
Primary_surgery, *n* (%)	
No	5011 (83.2)
Yes	1015 (16.8)

**Table 2:** Annual Percent Change by Sex and Race/Ethnicity.

Gender	APC	*p*-Value	95% CI
Male	0.9	<0.05	0.5, 1.2
Female	2.2	<0.05	1.7, 2.7
Male, Female (combined)	1.5	<0.05	1.2, 1.8
Non-Hispanic White	1.4	<0.05	0.1, 1.9
Non-Hispanic Black	1.0	<0.05	0.0, 2.1
Hispanic	2.5	<0.05	1.6, 3.5
Other	2.5	<0.05	1.1, 4

## Data Availability

All original data is available upon request.

## Appendix A

**Table A1:** Treatment Patterns by Age Groups.

	<50 y/o (*N* = 7307)	50–69 y/o (*N* = 53,799)	>70 y/o (*N* = 62,511)	Total (*N* = 123,617)	*p*-Value
Beam radiation, *n* (%)	1332 (18.5%)	9540 (18.1%)	6908 (11.3%)	17,780 (14.7%)	<0.0001
Chemotherapy, *n* (%)	4499 (61.6%)	32,393 (60.2%)	22,522 (36.0%)	59,414 (48.1%)	<0.0001
Primary surgery, *n* (%)	1015 (16.8%)	4334 (9.5%)	3239 (5.7%)	8588 (7.9%)	<0.0001
